# Cytokines as cellular communicators

**DOI:** 10.1155/S0962935196000579

**Published:** 1996-12

**Authors:** R. Debets, H. F. J. Savelkoul

**Affiliations:** 1 Department of Immunology Erasmus University P.O. Box 1738 Rotterdam 3000 DR The Netherlands

## Abstract

Cytokines and their receptors are involved in the pathophysiology of many diseases. Here we present a detailed review on cytokines, receptors and signalling routes, and show that one important lesson from cytokine biology is the complex and diverse regulation of cytokine activity. The activity of cytokines is controlled at the level of transcription, translation, storage, processing, posttranslational modification, trapping, binding by soluble proteins, and receptor number and/or function. Translation of this diverse regulation in strategies aimed at the control of cytokine activity will result in the development of more specific and selective drugs to treat diseases.


Mediators of Inflammation, 5, 417–423 (1996)

### Cytokines as cellular communicators

R. Debets and H. F. J. Savelkoul[CA]

Department of Immunology, Erasmus University, P.O. Box 1738, 3000 DR Rotterdam, The Netherlands

[CA]Corresponding Author

**CYTOKINES and their receptors are involved in the pathophysiology of many diseases. Here we present a detailed review on cytokines, receptors and signalling routes, and show that one important lesson from cytokine biology is the complex and diverse regulation of cytokine activity. The activity of cytokines is controlled at the level of transcription, translation, storage, processing, post-translational modification, trapping, binding by soluble proteins, and receptor number and/or function. Translation of this diverse regulation in strategies aimed at the control of cytokine activity will result in the development of more specific and selective drugs to treat diseases.**

**Key words:** Control of activity, Cytokine, Interference in cytokine activity, Receptor, Signalling routes

### Introduction

For over two decades, it has been recognized that immune-competent cells produce peptide mediators, now termed cytokines. Cytokines act as chemical communicators between cells, but mostly not as effector molecules in their own right. The biological function of many cytokines is mediated through their ability to regulate gene expression in target cells. Recent developments have made it clear that cytokines play crucial roles in the regulation of normal growth and development, protection against inflammation and infection, neoplastic transformation, wound-healing and angiogenesis. For example, inflammatory mediators are involved in the pathophysiology of septic shock and they are often associated with disease severity. In such diseases the balance between pro-inflammatory cytokines (interleukin-1 (IL-1), IL-6, IL-8, tumour necrosis factor-alpha (TNF-α)) and anti-inflammatory compounds (IL-1 receptor antagonist (IL-1ra), IL-10, soluble IL-1 receptor (sIL-1R), sTNFR) determines to a large extent the final outcome.[1] Also, in many diseases it was shown that production levels or activity of important cytokines is perturbed. Understanding the control of cytokine activity is therefore important as it may provide new potential strategies to treat diseases.

In general, cytokines are not expressed constitutively, but are rapidly produced after stimulation and act locally to restore homeostasis. Cytokines bind with high affinity to specific receptors on the surface of target cells. When cytokine receptors are expressed on the cytokine-producing cell, autocrine cellular activation may be the result. If the receptor is expressed on a neighbouring cell, binding may result in paracrine cytokine activation. These features could explain the sometimes high local concentration and profound local effects of cytokines. A characteristic feature of cytokines is their functional pleiotropy (cytokines result in diverse effects upon binding to different target cells) and redundancy (several cytokines can take over each other's effects), which can be largely explained at the molecular level of receptor systems (see below).

### Families of Cytokines

Table 1 presents a scheme in which cytokines are grouped according to their three-dimensional structure and receptor usage.[2–4] This scheme is still incomplete and inconclusive. At least six different families of cytokines have been identified: the haemopoietins (four α-helical bundles); IL-1 and fibroblast growth factor (FGF) (capped β-barrel of 12 antiparallel β-strands); TNF (sandwich of antiparallel β-sheets in jelly roll motif); transforming growth factor-alpha (TGF-α) and epidermal growth factor (EGF) (β-sheets); TGF-β, platelet-derived

© 1996 Rapid Science Publishers

**Table 1.** Structural families of cytokines and their receptors

| Families of cytokines[a] | Members | Superfamilies (SF) of cytokine receptors |
|---|---|---|
| Haemopoietins[b] | | Cytokine receptor SF class I |
| | IL-6, IL-11, LIF, OM, CNTF, CT-1 | gp130 |
| | IL-3, IL-5, GM-CSF | common β-chain |
| | IL-2, IL-4, IL-7, IL-9, IL-13, IL-15 | common γ-chain |
| | IL-10, IFN-α, IFN-β, IFN-γ | Cytokine receptor SF class II |
| | M-CSF | Immunoglobulin SF |
| IL-1 | IL-1α, IL-1β, IL-1ra, FGF-α, FGF-β | Immunoglobulin SF |
| TNF | TNF-α, TNF-β, LT-β | TNF receptor SF |
| TGF-α | TGF-α, EGF | Protein tyrosine kinase receptor SF |
| TGF-β | TGF-β1, TGF-β2, TGF-β3 | TGF-β receptors[c] |
| | PDGF, VEGF | Immunoglobulin SF |
| | NGF | TNF receptor SF |
| Chemokines[d] | | Chemokine receptor SF |
| α subfamily | IL-8, GRO-α, GRO-β, GRO-γ, ENA-78, IP-10, MIG, GCP-2, PBP (and its cleavage products: CTAP-III, β-thromboglobulin, NAP-2), PF-4, SDF-1α, SDF-1β | |
| β subfamily | MCP-1, MCP-2, MCP-3, RANTES, I-309, MIP-1α, MIP-1β | |

[a]The families of cytokines are named after a representative member (except for the haemopoietins and chemokines).
[b]The family of haemopoietins also comprise G-CSF and erythropoietin (Epo), but data on their receptors are insufficient for receptor classification.
[c]To date, the TGF-β receptors are not grouped into a SF of cytokine receptors.
[d]Abbreviations (apart from those explained in the text): CTAP, connective tissue activating protein; ENA, epithelial-derived neutrophil attractant; GCP, granulocyte chemotactic protein; IP, IFN-γ-inducible protein; LT, lymphotoxin; MIG, manokine induced by IFN-γ: MIP, macrophage inflammatory protein; NAP, neutrophil-activating peptide; PBP, platelet basic protein; PF, platelet factor; RANTES, regulated on activation, normal T-cell expressed and secreted; SDF, stromal cell-derived factor.

growth factor (PDGF), vascular endothelial growth factor (VEGF) and nerve growth factor (NGF) (cysteine knots); and chemotactic cytokines (chemokines) (triple-stranded, anti-parallel β-sheets in Greek key motif).[2–4] The large family of chemokines can be subdivided into at least α-chemokines (the two most amino-proximal cysteine residues are separated by an intervening amino acid: CXC) and β-chemokines (CC), which are functionally distinct, as they predominantly attract neutrophils and monocytes, respectively.[5] It is intriguing that different structural domains of cytokines (e.g. IL-1β) may display different or even antagonistic cytokine activities.[6]

### Families of Cytokine Receptors

Cytokine receptors are classified into several superfamilies based on common homology regions. The main superfamilies recognized today are the cytokine receptor superfamilies class I and class II, the immunoglobulin superfamily, the TNF receptor superfamily, the protein tyrosine kinase receptor superfamily, and the chemokine receptor superfamily.[3] Figure 1 illustrates schematically these cytokine receptor superfamilies. It should be noted that the superfamilies are not restricted to cytokine receptors (for example, several hormone receptors and leukocyte membrane polypeptides also belong to cytokine receptor superfamilies) and several cytokine receptors belong to more than one superfamily (e.g. IL-6 receptor belongs to both the class I receptor and immunoglobulin superfamilies). All cytokine receptors are single transmembrane proteins, except for the chemokine receptors which contain seven transmembrane domains.

The cytokine receptor superfamily class I receptors are characterized by the presence of combinations of cytokine and fibronectin III domains in the extracellular regions. The amino terminal cytokine domain(s) contains four conserved cysteine residues, and (one of) the membrane proximal fibronectin III domains contains the Trp-Ser-X-Trp-Ser (WSXWS) motif required for ligand binding.[7] Functional class I receptors consist of a complex of two or three receptor chains. These multisubunit receptors can be grouped according to the identity of common chains which are required for high affinity ligand binding and signal transduction. Three subgroups of class I receptors were identified sharing either gp130 (a glycoprotein with a relative molecular mass ($M_r$) of 130 kDa, cluster of differentiation (CD) 130), the common β-chain ($M_r$: 120 kDa, KH97) or the common γ-chain ($M_r$: 64-kDa)[8] (see Table 1 and Fig. 1). The gp130 chain is shared by the receptors for IL-6, IL-11, ciliary neurotrophic factor (CNTF), leukaemia inhibitory factor (LIF), oncostatin M (OM) and cardiotrophin-1 (CT-1).[9] Ligand binding by this group of receptors induces homodimerization of gp130 (in case of IL-6R and IL-11R) or heterodimerization of

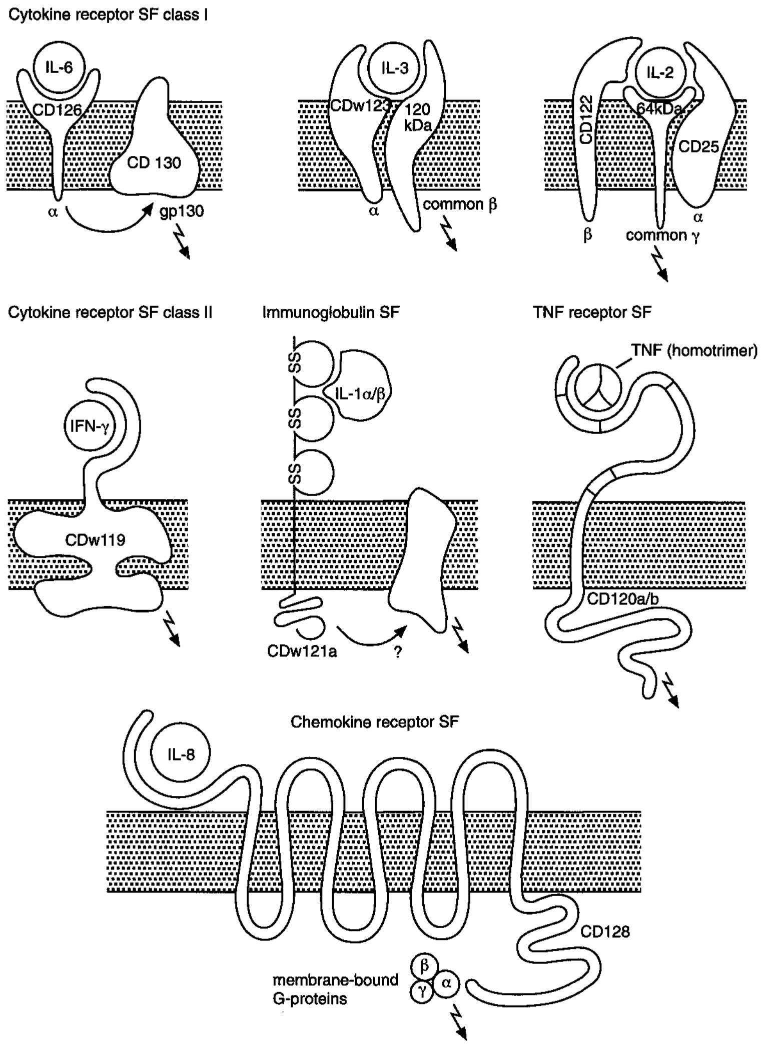

FIG. 1. Superfamilies of cytokine receptors. Examples are depicted, containing a representative cytokine bound to its receptor. Note that signalling through the IL-6 receptor complex is preceded by gp130 homodimerization, and that IL-1 receptor signalling is characterized by a cascade of protein phosphorylation. For simplicity reasons, the protein tyrosine kinase receptor SF is not shown, as it only covers the interaction with the growth factors TGF-α and EGF. Abbreviations are explained in the text and Table 1.

gp130 and LIFR (in case of CNTFR). LIF, OM and CT-1 are believed to directly induce heterodimerization of gp130 and LIFR (OM and perhaps CT-1 also induce heterodimerization of gp130 and the ligand-specific receptor). The LIFR, granulocyte-colony stimulating factor (G-CSF) R and IL-12R are highly homologous with gp130 and belong to the so-called gp130 family. The common β-chain is shared by IL-3R, IL-5R and granulocyte and macrophage-CSF (GM-CSF) R, whereas the common γ-chain is shared by IL-2R, IL-4R, IL-7R, IL-9R, IL-13R and IL-15R. Recent evidence suggests that the cytoplasmic parts of these signal transducing chains harbour two functional domains: a membrane-proximal region which is essential for proliferation signals (*c-myc* induction), and a membrane-distal region which is essential for differentiation signals (*c-fos* and *c-jun* induction).[10,11] The signalling routes which result from activation of the common chains are discussed below.

The cytokine receptor superfamily class II receptors (interferon (IFN) R and IL-10R) contain one or two extracellular fibronectin III domains, which lack the WSXWS motif characteristic of the class I receptors.[7] Ligand binding may induce receptor homodimerization (e.g. IFN-γR) and activate the Janus kinases (JAK)–signal transducers and activators of transcription (STAT) pathway (see below). The immunoglobulin superfamily receptors (IL-1R, IL-6R, FGFR, M-CSFR, PDGFR, VEGFR, stem cell factor R (SCFR)) contain one or more domains with the characteristic immunoglobulin fold.[12] Receptors belonging to the protein tyrosine kinase receptor superfamily (EGFR, insulin-like growth factor (IGF) R, FGFR, M-CSFR, PDGFR, VEGFR, SCFR) are in general growth factor receptors with an intracellular tyrosine kinase catalytic domain.[13] Ligand binding by these receptors induces receptor homo- or oligomerization, autophosphorylation and subsequent signalling. Members of the TNF receptor superfamily (TNFR and NGFR) are characterized by three or four cysteine-rich repeats in the extracellular part of the molecule.[14] These receptors may bind more than one ligand. Little is known about the mode of signal transduction by receptors of this superfamily. Receptors of the chemokine receptor superfamily are coupled to guanosine triphosphate (GTP) binding proteins which, after ligand binding, activate lipases, kinases, phosphatases and ion channels.[15]

**Table 2.** Selective usage of JAK kinases and STAT factors by cytokine receptors[a]

| Receptor | JAK kinases | STAT factors |
|---|---|---|
| IL-2 | 1, 3 | 3, 5 |
| IL-3 | 1, 2 | 5 |
| IL-4 | 1, 3 | 6 |
| IL-5 | 1, 2 | 5 |
| IL-6 | 1, 2, Tyk2 | 1, 3 |
| IL-7 | 1, 3 | 5 |
| IL-9 | 1, 3 | 3 |
| IL-10 | 1, Tyk2 | 1, 3 |
| IL-11 | 1, 2, Tyk2 | not known |
| IL-12 | 2, Tyk2 | 3, 4 |
| IL-13 | not known | 6 |
| IL-15 | 1, 3 | 5 |
| IFN-α/β | 1, Tyk2 | 1, 2, 3 |
| IFN-γ | 1, 2 | 1 |
| G-CSF | 2 | not known |
| GM-CSF | 1, 2 | 5 |
| CNTF | 1, 2, Tyk2 | 3 |
| OM | 1, 2, Tyk2 | 3 |
| LIF | 1, 2, Tyk2 | 3 |

[a]Modified from Refs 16, 17 and Schreiber RD, oral presentation, cytokine meeting, 6–10 October 1996, Geneva, Switzerland.

### Cytokine Receptor Signalling

Recent studies have demonstrated that the cytoplasmic domains of cytokine receptors are critical for signal transduction. Ligand-induced receptor di- or oligomerization typically results in activation of (receptor-associated) protein tyrosine kinases (such as members of the SRC kinases (JAK)) which are associated with the cytoplasmic tails of cytokine receptors. Subsequently, activated JAK1 are thought to phosphorylate several substrates (such as the receptors themselves, members of the RAS-mitogen activated protein (MAP) kinase cascades, and transcription factors). Phosphorylation of tyrosine-based motifs in the cytoplasmic domains of the receptors is followed by specific docking and activation of members of the STAT family of proteins.[15,16] Activated STATs form homodimers, translocate into the nucleus, and bind to specific responsive elements to initiate gene transcription. This JAK-STAT pathway results in rapid and specific transcriptional activation of target genes, and may, at least in part, contribute to cytokine-specific responses through selective activation of individual JAK kinases (JAK1, JAK2, JAK3 and TYK2) and STAT factors (STAT1 through STAT6).[17,18] Table 2 presents more details on receptor-mediated activation of induced JAK kinases and STAT factors for various cytokine receptors. Besides this tyrosine kinase pathway, also the serine kinase pathway is involved in cytokine-receptor mediated signalling. In activated T-cells the activation of transcription factors, like activating protein-1 (AP-1) (fos/jun dimers), members of the nuclear factor in activated T cells (NF-AT) family and NF-κB, play distinct roles in the induced differentiation. In the promotors of cytokine genes distinct combinations of these transcription factors determine the transcriptional activity of these loci. Activation of the individual cytokine genes requires binding of distinct sets of transcription factors. The data on the involvement of the JAK/STAT pathway do not preclude that the activation of these genes may be secondary to the activation of the above mentioned combination of transcription factors.

### Soluble Cytokine Receptors

It seems that essentially all single transmembrane cytokine receptors exist as soluble forms.[19] Soluble receptors can be translated from differentially spliced pre-mRNA molecules lacking the transmembrane domain (e.g. IL-4R, IL-5R, IL-6R, IL-7R, IL-9R, GM-CSFR, G-CSFR, TGF-αR). However, the presence of mRNA for soluble receptors not always seems to represent protein synthesis. Soluble receptors can also be generated by proteolysis of cell surface receptors (e.g. IL-1R, IL-2Rα, IL-6R, gp130, PDGFR, NGFR, TNFR, TGF-βR) and by phospholipase action on the glycosyl-phosphatidylinositol (GPI) anchor of CNTFR. Proteolytic shedding may be controlled by protein kinase C and may involve a metalloprotease, since phorbol myristate acetate (PMA)-induced shedding of IL-6R, TNFR type I and II as well as membrane TNF-α is blocked by a metalloprotease inhibitor, which was first identified as TNF-α protease inhibitor (TAPI).[20,21] Soluble cytokine receptors are found in appreciable amounts in body fluids, although the exact cellular source and function of these proteins is not completely clear.

### Virokines

The immunological relevance of cytokines and (soluble) cytokine receptors is clearly illustrated by the observation that several pathogenic virus strains, particularly pox viruses, encode proteins which counteract the anti-viral cytokine activity during the host response.[22] Examples of these so-called virokines include a homologue of the host suppressive cytokine IL-10 encoded by Epstein-Barr virus[23] and several poxviral homologues of soluble IL-1R (sIL-1R), sTNFR and sIFNR, which prevent interaction of the ligands with cellular receptors.[22,24] In addition, herpes viruses and cytomegalovirus encode chemokine receptors which are expressed on the membrane of infected cells, but probably display an altered function.[25] Furthermore, intracellular viral proteins have been described that interfere with cytokine maturation and cytokine-mediated responses.[22]

### Cytokine Biology

The current view on cytokine biology is that of a network of cytokines with additive, synergistic and opposite effects, and inducers and inhibitors of the expression of cytokines and/or their receptors, which combine to give an overall biological (or clinical) response.[26] Cytokines are potent molecules, of which the activity is regulated at multiple levels.

First, stimuli are required to induce as well as to suppress the transcription of most cytokine genes.[3] For example, infectious or toxic agents, mechanical injury, inflammatory mediators, and cytokines themselves (e.g. IL-1, TNF, IFN-γ) are potent cytokine inducers, whereas glucocorticoids, prostaglandins and cytokines (e.g. IL-10

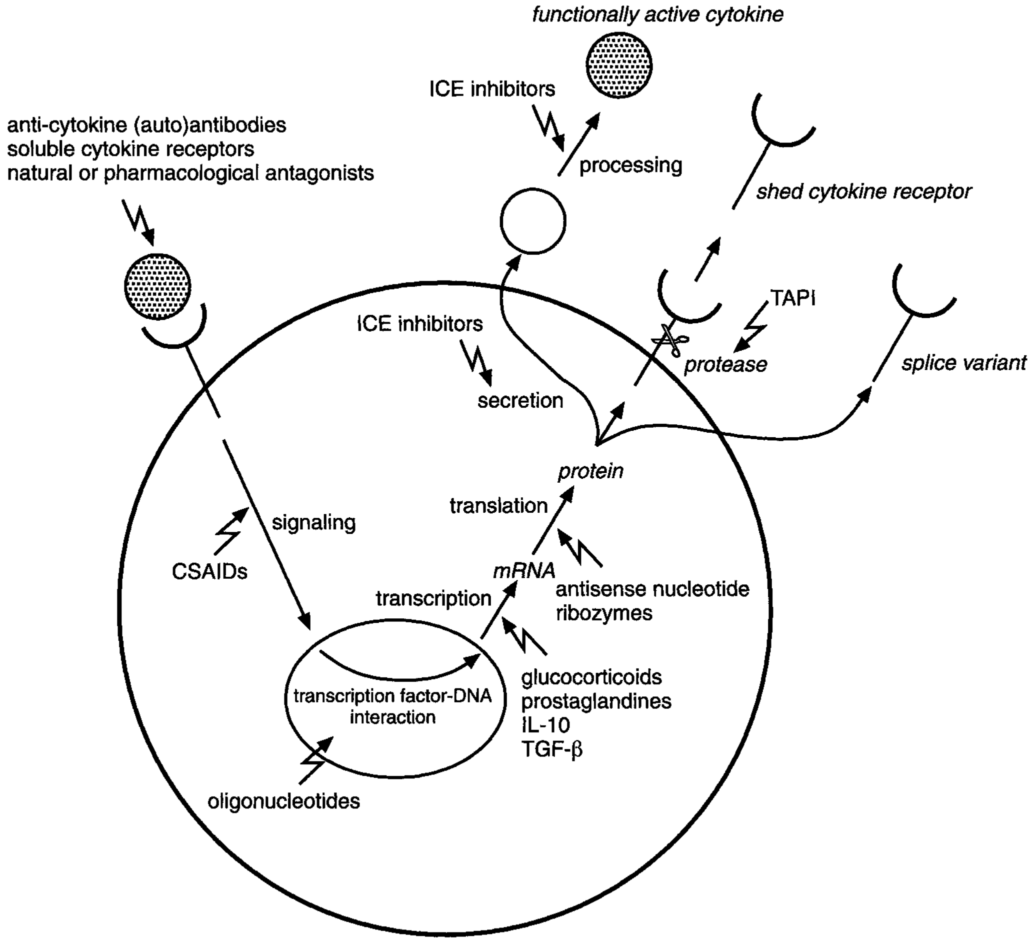

FIG. 2. Schematic outline of a target cell (represented only by a plasma and a nuclear membrane) illustrating the synthesis and activation of cytokines and cytokine receptors. At multiple sites interference can potentially lead to inhibition of production and/or activation of cytokines and their receptors. Numerous drugs have been reported which interfere with cytokine, receptor synthesis or activation. Those mentioned in the figure, therefore, merely serve as an example. Abbreviations (apart from those explained in the text): CSAID, cytokine synthesis anti-inflammatory drug; ICE, interleukin-1β converting enzyme.

and TGF-β) are potent inhibitors of cytokine gene expression. Note that genetic polymorphisms (e.g. IL-1α, IL-1ra and TNF-β[27–29]) may contribute to interindividual differences in cytokine expression upon stimulation.

Second, the extent of cytokine protein production is limited, since the consensus sequence UUAUUUAU in the 3′ end of most cytokine mRNAs promotes message instability.[30] Translation of cytokine mRNA transcripts may, in addition to the first signal, require an additional signal (e.g. in the case of IL-1β).[31]

Third, several cytokines are not released through the classic secretory pathway. These cytokines are produced as larger biologically inactive precursor molecules, which are stored within the cytoplasm (e.g. IL-1, IL-8, GM-CSF, TGF-β, PDGF) or expressed on the plasma membrane (e.g. TGF-α, IL-1α, TNF-α), and need to be proteolytically cleaved to release the mature molecule (e.g. TGF-α, IL-1α, IL-1β, TNF-α).[32–34] Extracellular enzymes may also contribute to the processing of precursor molecules (e.g. IL-1β, TGF-β).[35,36]

Fourth, post-translational modifications (e.g. cleavage-site of leader peptide, glycosylation, phosphorylation and/or multimerization) affect the biological activity of mature cytokines (e.g. IL-6, IL-8).[37–39]

Fifth, once secreted, several cytokines are trapped by cell surface binding proteins (e.g. TGF-β), extracellular matrix (e.g. LIF, IL-1, FGF), stromal cells (e.g. IL-3, IL-5, GM-CSF) and endothelial cells (e.g. IL-8).[3,40] It should be noted that the intracellular, membrane and extracellular pools of cytokines are available for rapid maturation and/or release in response to stimulation.

Sixth, soluble proteins which bind to cytokines can potentiate or antagonize cytokine activity. The proteins bind to cytokines either nonspecifically such as α2-macroglobulin[41] or specifically such as autoantibodies to cytokines (e.g. IL-1α, IL-6, IL-8, IL-10, monocyte chemotactic protein-1 (MCP-1), IFN-α)[42,43] and soluble cytokine receptors.[19] So far all known soluble forms of cytokine receptors were found to retain their ligand binding capacity. On the one hand, these soluble proteins may agonize cytokine activity by acting as chaperons, increasing the cytokine's persistence.[4,44] Moreover, in case of sIL-6R and sCNTFR, the soluble proteins form a biologically active binary complex with their ligands (IL-6—sIL-6R, CNTF—sCNTFR) which may act on cells only expressing gp130 on their cell surface, but not IL-6R or CNTFR (normally anticipated as cells being unresponsive to IL-6 or CNTF).[19] Note that in line with this 'trans-signalling pathway' is the fact that gp130 is present essentially on all cell types, whereas IL-6R and CNTFR are not expressed ubiquitously. On the other hand, soluble proteins may antagonize cytokine activity by acting as scavengers of cytokines (by competing with membrane receptors), by neutralizing their activity, by enhancing antibody-dependent cell-mediated cytotoxicity, by preventing extravascular escape and by promoting excretion.[4] Whether soluble proteins will act as agonists or antagonists of cytokine activity is, at least in part, determined by the ratio between membrane receptors and soluble proteins: the predominance of membrane receptors favours agonistic activity, whereas the predominance of soluble proteins favours antagonistic activity.[44]

Finally, cytokine activity can be regulated by modulating the number and/or function of membrane receptors. Receptor number may be modulated by controlling gene expression, internalization or the generation of soluble receptors. Receptor affinity and/or function may be modulated by affecting receptor phosphorylation and/or glycosylation, by competition for common receptor chains or signal transduction molecules[16,41] or by binding cytokine receptor antagonists. These latter molecules bind to the receptor but do not cause signal transduction (for example, IL-1ra, IL-4δ2 and $(p40)_2$ antagonize IL-1, IL-4 and IL-12 functions, respectively).[45–47]

Strategies aiming at controlling cytokine activity take this into account and are schematically illustrated in Fig. 2.

### References

1. Kornelisse RF, Hazelzet JA, Savelkoul HFJ, Hop WCJ, Suur MH, Borsboom ANJ, Risseeuw-Appel IM, Van Der Voort E, De Groot R. The relationship between plasminogen activator inhibitor-1 and proinflammatory mediators in children with meningococcal septic shock. *J Infect Dis* 1996; **173**: 1148–1156.
2. Boulay JL, Paul WE. The interleukin-4 family of lymphokines. *Curr Opin Immunol* 1993; **3**: 573–581.
3. Callard R, Gearing A. *The Cytokine Factsbook*. London: Academic Press, 1994; 12–27.
4. Debets R, Savelkoul HFJ. Cytokine antagonists and their potential therapeutic use. *Immunol Today* 1993; **15**: 455–458.
5. Baggiolini M, Dewald B, Moser B. IL-8 and related chemotactic cytokines-CXC and CC chemokines. *Adv Immunol* 1993; **55**: 97–179.
6. Boraschi D, Nencioni L, Villa L, Censini S, Bossù P, Ghiara P, Presentini R, Perin F, Frasca D, Doria G, Forini G, Musso T, Giovarelli M, Ghezzi P, Bortini F, Besedovsky HO, Del Rey A, Sipe JO, Antoni G, Silvesta S, Tagliabue A. In vivo stimulation and restoration of the immune response by the non-inflammatory fragment 163-171 of human interleukin 1β. *J Exp Med* 1988; **168**: 675–686.
7. Bazan JF. Structural design and molecular evolution of a cytokine receptor superfamily. *Proc Natl Acad Sci USA* 1990; **87**: 6934–6938.
8. Kishimoto T, Taga T, Akira S. Cytokine signal transduction. *Cell* 1994; 76: 253–262.
9. Kishimoto T, Akira S, Narazaki M, Taga T. Interleukin-6 family of cytokines and gp130. *Blood* 1995; **86**: 1243–1254.
10. Murakami M, Narazaki M, Hibi M, Yawata H, Yasukawa K, Hamaguchi M, Taga T, Kishimoto T. Critical cytoplasmic region of the IL-6 signal transducer, gp130, is conserved in the cytokine receptor family. *Proc Natl Acad Sci USA* 1991; **88**: 11349–11353.

11. Ihle JN, Witthuhn BA, Quelle F, Yamamoto K, Silvennoinen O. Signaling through the hematopoietic cytokine receptors. *Annu Rev Immunol* 1995; **13**: 369–398.
12. Amzel LM, Poljak RJ. Three-dimensional structure of immunoglobulins. *Annu Rev Biochem* 1979; **48**: 961–967.
13. Ullrich A, Schlessinger J. Signal transduction by receptors with tyrosine kinase activity. *Cell* 1990; **61**: 203–212.
14. Mallett S, Barclay AN. A new superfamily of cell surface proteins related to the nerve growth factor receptor. *Immunol Today* 1991; **12**: 220–223.
15. Gerard C, Gerard NP. The pro-inflammatory seven-transmembrane segment receptors of the leucocyte. *Curr Opin Immunol* 1994; **6**: 140–145.
16. Schindler C, Darnell JE. Transcriptional responses to polypeptide ligands: the JAK-STAT pathway. *Annu Rev Biochem* 1995; **64**: 621–651.
17. Ivashkiv LB. Cytokines and STATs: how can signals achieve specificity? *Immunity* 1995; **3**: 1–4.
18. Ihle JN. Cytokine receptor signalling. *Nature* 1995; **377**: 591–594.
19. Rose-John S, Heinrich PC. Soluble receptors for cytokines and growth factors: generation and biological function. *Biochem J* 1994; **300**: 281–290.
20. Crowe PD, Walter BN, Mohler KM, Otten-Evans C, Black RA, Ware CF. A metallo-protease inhibitor blocks shedding of the 80-kD TNF receptor and TNF processing in T lymphocytes. *J Exp Med* 1995; **181**: 1205–1210.
21. Müllberg J, Durie FH, Otten-Evans C, Alderson MR, Rose-John S, Cosman D, Black RA, Mohler KM. A metalloprotease inhibitor blocks shedding of the IL-6 receptor and the p60 TNF receptor. *J Immunol* 1995; **155**: 5198–5205.
22. Alcamí A, Smith GL. Cytokine receptors encoded by pox viruses: a lesson in cytokine biology. *Immunol Today* 1995; **16**: 474–478.
23. Hsu D-H, De Waal Malefyt R, Fiorentino DF, Dang MN, Vieira P, De Vries J, Spits H, Mosmann TR, Moore KW. Expression of interleukin-10 activity by Epstein-Barr virus protein BCRF1. *Science* 1990; **250**: 830–832.
24. Spriggs MK, Hruby DE, Maliszewski CR, Pickup DJ, Sims JE, Buller RM, Van Slyke J. Vaccinia and cowpox viruses encode a novel secreted interleukin-1 binding protein. *Cell* 1992; **71**: 153–167.
25. Ahuja SK, Murphy PM. Molecular piracy of mammalian interleukin-8 receptor type B by herpesvirus saimiri. *J Biol Chem* 1993; **268**: 20691–20694.
26. Chatenoud L. Allograft rejection: the role of the cytokine network. *Eur Cyt Network* 1992; **3**: 509–513.
27. Ruddle NH. Tumor necrosis factor (TNF-α) and lymphotoxin (TNF-β). *Curr Opin Immunol* 1992; **4**: 327–332.
28. Baily S, Di Giovine FS, Blakemore AI, Duff GW. Genetic polymorphism of human interleukin-1 alpha. *Eur J Immunol* 1993; **23**: 1240–1245.
29. Tarlow JK, Blakemore AI, Lennard A, Solari R, Hughes HN, Steinkasserer A, Duff GW. Polymorphism in human IL-1 receptor antagonist gene intron 2 is caused by variable numbers of an 86 bp tandem repeat. *Hum Genet* 1993; **91**: 403–404.
30. Cosman D. Control of messenger RNA stability. *Immunol Today* 1987; **8**: 16–17.
31. Schindler R, Clark BD, Dinarello CA. Dissociation between interleukin-1β mRNA and protein synthesis in human peripheral blood mononuclear cells. *J Biol Chem* 1990; **265**: 10232–10237.
32. Massague J, Pandiella A. Membrane-anchored growth factors. *Annu Rev Biochem* 1993; **62**: 515–541.
33. Howard AD, Kostura MJ, Thornberry N, Ding G-JF, Limjuco G, Weidner J, Salley JP, Hogquist KA, Chaplin DD, Mumford RA, Schmidt JA, Tocci MJ. IL-1-converting enzyme requires aspartic acid residues for processing of the IL-1β precursor at two distinct sites and does not cleave 31-kDa IL-1α. *J Immunol* 1991; **147**: 2964–2969.
34. Schröder JM. Cytokine networks in the skin. *J Invest Dermatol* 1995; **105**: 20S–24S.
35. Hazuda DJ, Strickler J, Kueppers F, Simon PL, Young PR. Processing of precursor interleukin 1 beta and inflammatory disease. *J Biol Chem* 1990; **265**: 6318–6322.
36. Harpel JG, Metz CN, Kojima S, Rifkin DB. Control of transforming growth factor-beta activity: latency vs. activation. *Prog Growth Factor Res* 1993; **4**: 321–335.
37. Seghal PB. Interleukin 6: molecular pathophysiology. *J Invest Dermatol* 1990; **94**: 2S–6S.
38. Van Damme J, Van Beeumen J, Conings R, Decock B, Billiau A. Purification of granulocyte chemotactic peptide/interleukin-8 reveals N-terminal sequence heterogeneity similar to that of beta-thromboglobulin. *Eur J Biochem* 1989; **81**: 337–344.
39. Opdenakker G, Rudd PM, Wormald M, Dwek RA, Van Damme J. Cells regulate the activities of cytokines by glycosylation. *FASEB J* 1995; **9**: 453–457.
40. Tanaka Y, Adams DH, Shaw S. Proteoglycans on endothelial cells present adhesion-inducing cytokines to leucocytes. *Immunol Today* 1993; **14**: 111–115.
41. Cosman D. The hematopoietin receptor superfamily. *Cytokine* 1993; **5**: 95–106.
42. Bendtzen K, Svenson M, Jonsson V, Hippe F. Autoantibodies to cytokines—friends or foes? *Immunol Today* 1990; **11**: 167–169.
43. Sylvester I, Suffredini AF, Boujoukos AJ, Martich GD, Danner RL, Yoshimura T, Leonard EJ. Neutrophil attractant protein-1 and monocyte chemoattractant protein-1 in human serum. Effects of intravenous lipopolysaccharide on free attractants, specific IgG autoantibodies and immune complexes. *J Immunol* 1993; **151**: 3292–3298.
44. Klein B, Brailly H. Cytokine-binding proteins: stimulating antagonists. *Immunol Today* 1995; **16**: 216–220.
45. Eisenberg SP, Evan RJ, Arend WP, Verderber E, Brewer MT, Hannum CH, Thompson RC. Primary structure and functional expression from complementary DNA of a human interleukin-1 receptor antagonist. *Nature* 1990; **343**: 341–346.
46. Atamas SP, Choi J, Yurovsky VV, White B. An alternative splice variant of human IL-4, IL-4δ2, inhibits IL-4-stimulated T cell proliferation. *J Immunol* 1996; **156**: 435–441.
47. Ling P, Gately MK, Gubler U, Stern AS, Lin P, Hollfelder K, Su C, Pan YC, Hakimi J. Human IL-12 p40 homodimer binds to the IL-12 receptor but does not mediate biologic activity. *J Immunol* 1995; **154**: 116–127.

**Received 25 September 1996; accepted 14 October 1996**
